# A Non-Invasive Nomogram for Preoperative Prediction of Microvascular Invasion Risk in Hepatocellular Carcinoma

**DOI:** 10.3389/fonc.2021.745085

**Published:** 2021-12-24

**Authors:** Huanhuan Wang, Ye Lu, Runkun Liu, Liang Wang, Qingguang Liu, Shaoshan Han

**Affiliations:** Department of Hepatobiliary Surgery, The first Affiliated Hospital of Xi’an Jiaotong University, Xi’an, China

**Keywords:** hepatocellular carcinoma, microvascular invasion, VEGF-A, nomogram, prediction model

## Abstract

**Background:**

Microvascular invasion (MVI) is a significant predictive factor for early recurrence, metastasis, and poor prognosis of hepatocellular carcinoma. The aim of the present study is to identify preoperative factors for predicting MVI, in addition to develop and validate non-invasive nomogram for predicting MVI.

**Methods:**

A total of 381 patients with resected HCC were enrolled and divided into a training cohort (*n* = 267) and a validation cohort (*n* = 114). Serum VEGF-A level was examined by enzyme-linked immunosorbent assay (ELISA). Risk factors for MVI were assessed based on univariate and multivariate analyses in the training cohort. A nomogram incorporating independent risk predictors was established and validated.

**Result:**

The serum VEGF-A levels in the MVI positive group (n = 198) and MVI negative group (n = 183) were 215.25 ± 105.68 pg/ml and 86.52 ± 62.45 pg/ml, respectively (*P* <0.05). Serum VEGF-A concentration ≥138.30 pg/ml was an independent risk factor of MVI (OR: 33.088; 95%CI: 12.871–85.057; *P <*0.001). Higher serum concentrations of AFP and VEGF-A, lower lymphocyte count, peritumoral enhancement, irregular tumor shape, and intratumoral artery were identified as significant predictors for MVI. The nomogram indicated excellent predictive performance with an AUROC of 0.948 (95% CI: 0.923–0.973) and 0.881 (95% CI: 0.820–0.942) in the training and validation cohorts, respectively. The nomogram showed a good model fit and calibration.

**Conclusions:**

Higher serum concentrations of AFP and VEGF-A, lower lymphocyte count, peritumoral enhancement, irregular tumor shape, and intratumoral artery are promising markers for MVI prediction in HCC. A reliable non-invasive nomogram which incorporated blood biomarkers and imaging risk factors was established and validated. The nomogram achieved desirable effectiveness in preoperatively predicting MVI in HCC patients.

## Introduction

Hepatocellular carcinoma(HCC)is the sixth most commonly diagnosed cancer and the fourth leading cause for cancer-related deaths worldwide (1). Currently, surgical resection and liver transplantation are the potentially curative treatments for HCC patients at early stage (2, 3). The poor prognosis of HCC patients is largely due to the high frequency of tumor recurrence and metastasis after surgical treatments (4, 5). Of note, microvascular invasion is an independent risk factor for early recurrence, metastasis, and poor prognosis of HCC (6, 7).

Microvascular invasion (MVI) refers to the presence of cancer cells that infiltrated into the surrounding blood vessels lined with endothelial cells under the microscopic observation (8). Currently, postoperative pathology is the gold standard for diagnosis of MVI. Given that MVI status may influence the choice of treatment, it is necessary to develop an accurate predictive model of MVI based on available factors. The non-invasive diagnostic model of MVI can indicate the risk of MVI presence and enable surgeons to adopt appropriate surgical procedures for HCC patients. For example, an anatomical resection should be recommended when the patient with high risk of MVI (9). For HCC patient identified with MVI positive, tumors should be surgically resected with wide margins (10). In addition, patients with high risk of MVI are suitable candidates for adjuvant TACE (11). In recent years, several studies tried to establish predictive models for preoperative estimation of MVI. Serum alpha-fetoprotein and PIVKA-II (12) were reported as possible markers for predicting MVI. Imaging features were proposed as preoperative predictors of MVI, such as tumor size (13), non-smooth tumor margin, and peritumoral enhancements (14). Angiogenesis is an essential event in tumor growth, invasion, and metastasis (15). Vascular endothelial growth factor A (VEGF-A) can induce angiogenesis by promoting proliferation, migration, and tube formation of endothelial cells (16). VEGF-A contributes to tumor growth and metastasis by enhancing tumor angigenesis and vascular permeability (17). VEGF-A has been reported to be a critical molecular marker to predict MVI (18), and increased VEGF-A expression correlates significantly with faster tumor growth rate and intrahepatic and distant metastases (19).

Recenty, several studies have tried to build a nomogram model for for predicting MVI status,which showed good sensitivity and specificity; however, there are still some limitations. For example, Wei et al. (20) used radiomics analysis to develop a deep learning model, which focused solely on computed tomography (CT) and gadoxetic acid-enhanced magnetic resonance imaging (EOB-MRI). Banerjee et al. (21) mapped CT image features to HCC-specific vascular invasion gene expression to predict histological MVI with high degree of accuracy. However, technological complexity and high costs of multi-gene expression assays made these methods difficult to apply in routine clinical setting. Nitta et al. (22) developed a predictive model for MVI of HCC; however, imaging features were only extracted inside the tumor. A more effective evaluation should also focus on the radiomic features at the tumor periphery. Ryu et al. (23) built a clinical scoring system for predicting MVI, but had too small sample sizes (n = 120) to provide precise estimates of sensitivity and specificity. Thus, it is necessary to establish a readily accessible and effective model to predict MVI status and MVI risk of HCC.

The present study aimed to analyze the imaging, clinical, and pathological features of HCC patients by retrospective analysis. The univariate analysis was used to assess the single factor for discriminating MVI in the training cohort. The significant variables obtained from univariate analysis were entered into multivariate logistic regression analysis to determine potential risk factors of MVI. Moreover, the data analysis software (R-studio 4.0.3) was used to build a non-invasive nomogram for prediction of MVI in HCC patients. This nomogram combined serum VEGF-A, AFP, lymphocyte count, and imaging features, especially radiomic features extracted from contrast-enhanced CT. Finally, we carried out an internal validation to assess the predictive performance of nomogran model, and to evaluate the clinical usefulness of the model.

## Methods and Materials

### Patients and Study Design

**Figure 1:**
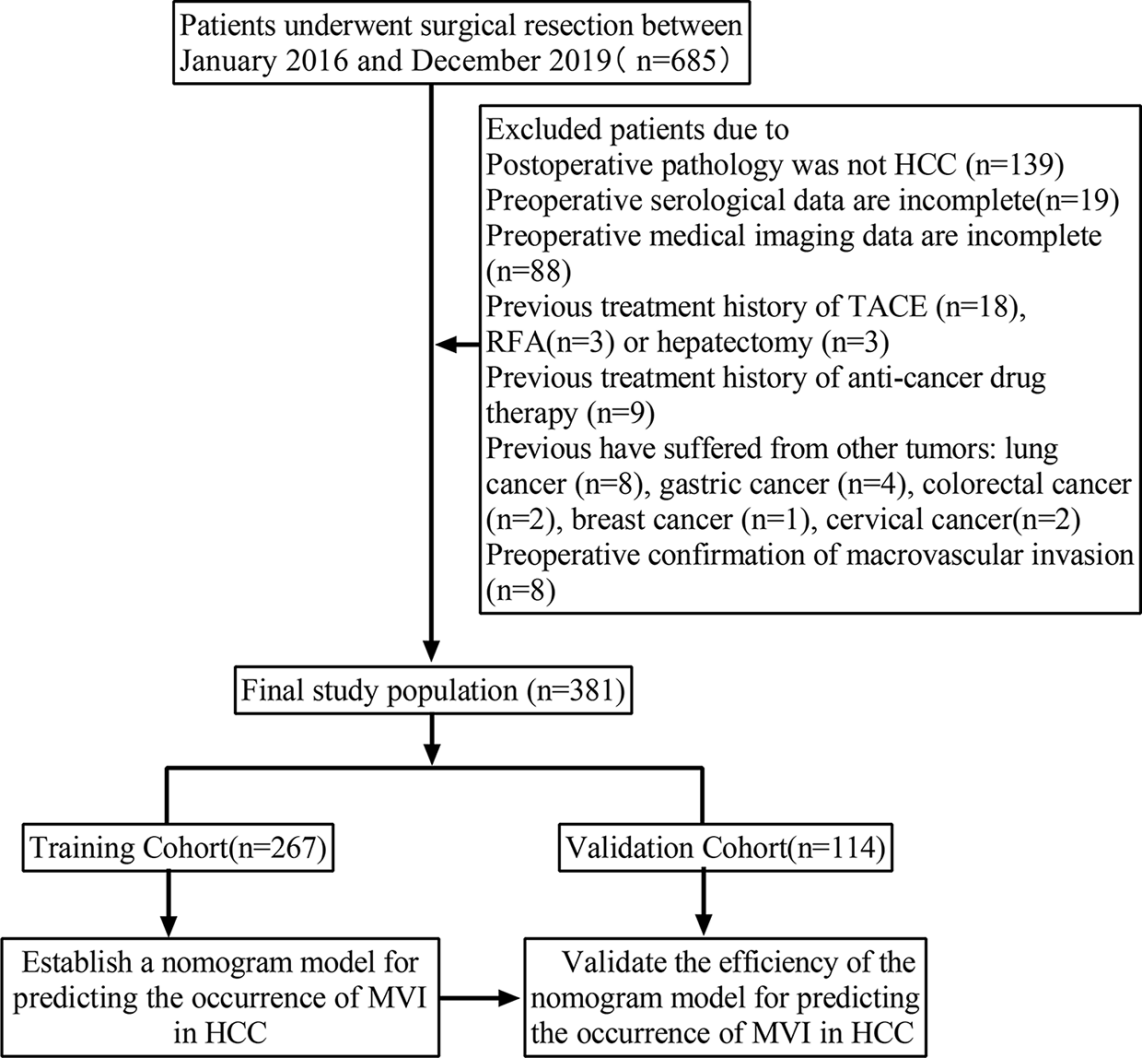
Flow chart of patients screening and grouping.

The selection procedure and study design are shown in **Figure 1**. A total of 685 patients who underwent curative hepatectomy in the First Affiliated Hospital of Xi’an Jiaotong University between January 2016 and December 2019 were included in this study. The major inclusion criteria are listed as follows: (1) diagnosis of HCC by pathologic criteria; (2) a specific diagnosis of the presence or absence of MVI; (3) complete preoperative hematological indicators and abdominal enhanced CT data were available; and (4) upper abdominal enhanced CT and blood examination performed less than 1 week prior to surgery. The major exclusion criteria are listed as follows: (1) preoperative treatment, such as trans-arterial chemoembolization (TACE), radiofrequency ablation, and antineoplastic agents; (2) the presence of other malignant tumors in the patient’s medical history; (3) the presence of signs of macrovascular invasion before partial hepatectomy, such as portal vein invasion or hepatic vein invasion. Finally, 381 patients with HCC were included and randomly divided into two groups: a training cohort (n = 267) and a validation cohort (n = 114) with a ratio of 7:3. The former was used to analyze the data and build the MVI non-invasive predictive model, and the latter was used to verify the reliability of the model. This retrospective study used anonymous data and was approved by the First Affiliated Hospital of Xi’an Jiaotong University.

### Basic Data of Patients and Blood Markers

Basic data of patients included the patient’s age, sex, and history of hepatitis and cirrhosis. The patients with hepatitis in the present study included viral hepatitis caused by HBV or HCV. All patients received routine laboratory examinations before curative resection, namely, routine blood test, blood biochemical examination, and tumor marker examination. The research indicators in this study included serum alpha-fetoprotein (AFP), aspartate aminotransferase (AST), alanine aminotransaminase (ALT), bilirubin, albumin, the count of neutrophils, platelets, and lymphocytes.

### Contrast-Enhanced CT and Imaging Data Analysis

All patients underwent an upper abdominal contrast-enhanced CT scan preoperatively. The scanning range was from the dome of diaphragm to the lower pole of both kidneys. Arterial-phase CT scans were obtained at 30 s, portal-phase CT scans were obtained at 60 s, and late-phase CT scans were obtained at 90–120 s. The tumor size, peritumoral enhancement, peritumoral boundary, tumor shape, intratumoral artery, and multiple tumors in enhanced CT images were analyzed by two senior radiologists. The two radiologists both kept ignorant of the clinicopathological information except for the diagnosis of HCC. In the case of inconsistent in assessment, they reached a consensus by means of reviewing and discussion, the final analysis was based on their consensus.

**Figure 2:**
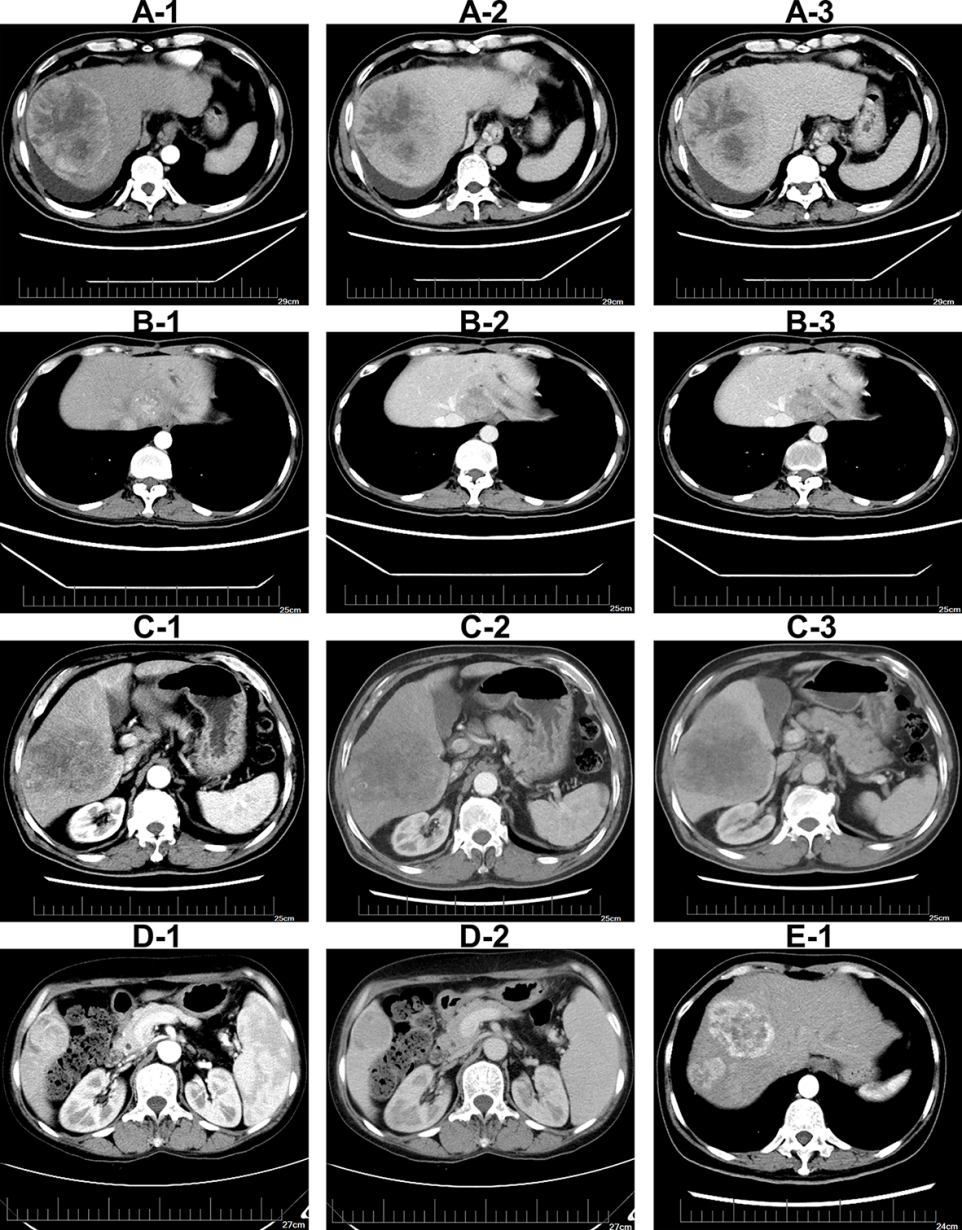
Illustrations of imaging features on contrast-enhanced CT imaging **(A-1–A-3)**. Tumor with peritumoral enhancement in arterial-phase, portal-phase, and late-phase **(B-1–B-3)**.Tumor with intratumoral artery in arterial-phase, portal-phase, and late-phase **(C-1–C-3)**.Tumor with irregular shape in arterial-phase, portal-phase and late-phase **(D-1, D-2)**. Tumor with unclear boundary in contrast-enhanced CT imaging. Tumor with multiple nodules in CT imaging **(E-1)**.

a) Tumor size was defined as the maximum diameter of the tumor measured by digital calipers on contrast-enhanced CT transverse images. When the number of tumor nodules was ≥2, the largest one was selected for diameter measurement.
b) Peritumoral enhancement was defined as detectable portion of crescent or irregular enhancement outside the tumor margin in arterial-phase. However, the enhancement part attenuated in portal-phase and late-phase (**Figures 2A1–3**).
c) Intratumoral artery was defined as continuous enhancement of arterial vessels observed in the tumor during the arterial phase, and the enhancement part attenuated in the portal-phase and late-phase (**Figures 2B1–3**).
d) Tumor shape was divided into regular shape and irregular shape. The regular shape of tumor was defined according to CT transverse images as round or oval. And irregular tumor shape included fusiform and other irregular profiles (**Figures 2C1–3**).
e) Tumor boundary was divided into clear boundary and unclear boundary. A clear one was defined as a sharp demarcation between tumor margin and normal liver tissue at any level on contrast-enhanced CT transverse images. Otherwise, it was defined as the unclear boundary of the tumor (**Figures 2D1, 2**).
f) Multiple tumors was defined as unfused multiple tumor nodules (≥2) showing obvious “fast in and out” sign
in contrast-enhanced CT images (**Figure 2E-1**).

### Pathological Examination and MVI Classification

All pathological specimens were independently reassessed by two pathologists. MVI was defined as the presence of a tumor cell in a portal vein, hepatic vein, or a large capsular vessel of the surrounding hepatic tissue lined with endothelium that was visible only on microscopy (24). Pathological sampling process was carried out in agreement with the practice guidelines for handling surgical specimens, and the sampling process included the sites, distances, and volumes for tissue sampling from HCC surgical specimens in order to make more accurate pathological diagnoses, including the presence of MVI (**Supplementary Figure 1**). Given the diagnosis of MVI is related to the distribution and volumes of the tumor tissue sampling for liver specimens. The classification of MVI is defined as follows: M0, no MVI; M1 (low-risk), the number of MVI is ≤5 and at a distance of ≤1 cm away from the tumor capsule; M2 (high-risk), the number of MVI is >5 and at a distance of >1 cm from the tumor capsule (25). The degree of differentiation of tumor cells described according to the 2019 WHO classification (26).

### Enzyme-Linked Immunosorbent Assay (ELISA)

Blood samples were obtained from the included patients prior to liver resection. The VEGF-A concentration in the blood was measured with a VEGF ELISA Kit (R & D Systems China Co. Ltd., Shanghai, China). All ELISA assays were performed in duplicates. The optimal threshold concentrations for predicting diagnosis of MVI and non MVI were determined through the analysis of the working characteristic curve of the subjects.

### Statistical Analysis

Statistical analyses were performed with SPSS 22.0 software (IBM, New York, USA). Continuous variables were presented as means ± standard deviations (SD). A normality test was performed using a Shapiro–Wilk test. When data were normally distributed, the Student’s t-test was used; otherwise the Mann–Whitney U test was performed to compare differences between groups. For a continuous variable, it was first converted to a categorical variable according to the clinical or reference range cut-off point. Categorical variables were compared using a χ^2^ test or the Fisher’s exact test. Univariate analysis was used to assess the significance of each variable for discriminating MVI presence in the training cohort. The stepwise multivariate logistic analysis was applied using variables with *P*-values <0.05 to identify independent predictors and to rule out potential confounding variables. All the risk factors that were significantly associated with MVI were subsequently entered in a final nomogram model.

Kappa statistics was used to test inter-observer agreement. Kappa value was calculated for evaluating inter-observer agreement of radiological features and histopathological features. Kappa value ≥0.75 indicated good consistency, 0.75 > kappa value > 0.4 was regarded as medium consistency, and kappa value <0.4 for poor consistency. All statistical tests were two-tailed, and *P*-value <0.05 was considered as statistically significant.

### Development and Validation of Nomogram

A nomogram was formulated to predict MVI based on the results of multivariate logistic regression analysis in the training cohort and by employing the *rms* package in R version 4.0.3 (http://www.r-project.org/). The scoring systems provided a numeric value, which indicated the risk of MVI presence. The ROC curve and AUC were used to assess the model’s ability to distinguish events and nonevents. The sensitivity, specificity, accuracy, positive predictive value, and negative predictive value were presented, as calculated below:


Sensitivity=True positive rate (TPR)=(Σ True positive)/(Σ Condition positive);

Specificity=True negative rate (TNR)=(Σ True negative)/(Σ Condition negative);

Accuracy=[(Σ True positive)+(Σ True negative)]/(Σ Total population);

Positive predictive value (PPV)=(Σ True positive)/(Σ Predicted condition positive);

Negative predictive value (NPV)=(Σ True negative)/(Σ Predicted condition negative).

To analyze the agreement between nomogram and actual observation, calibration curve of the nomogram for predicting MVI was performed by comparing predicted MVI status with actual MVI status. Meanwhile, the nomogram was subjected to 1,000 bootstrap resamples for internal validation. Each calibration plot consisted of an ideal line and an apparent curve. The apparent curve represented the fitting degree between the predicted results and the actual situation. While the diagonal line indicated a perfect match between nomogram-predicted probability of MVI presence (x-axis) and actual probability of MVI presence (y-axis). Hosmer–Lemeshow goodness of fit test was used to assess the model fit (Hosmer–Lemeshow statistic ≥0.05).

### Clinical Use of the Nomogram

Decision curve analysis (DCA) and Clinical impact curve (CIC) were applied to probe into the clinical usefulness of the nomogram. DCA and CIC were performed in the training and validation cohorts by employing the *rmda* package in R version 4.0.3 (http://www.r-project.org/). Graphical analysis of the net benefit against the threshold probability yielded a decision analysis curve, which could then be used to assess the net benefit of nomogram-assisted decisions at different threshold probabilities, compared with the net benefit of decisions made with the assumption that either all or no patient has the outcome of interest. The CIC was also conducted to determine the clinical usefulness of the nomogram in the training and validation sets, which quantified the net benefits at different threshold probabilities in the study.

## Results

### Clinical and Imaging Characteristics in the Training and Validation Cohorts

A total of 685 HCC patients underwent a radical (R0) partial hepatectomy in our center, and 381 qualified patients were enrolled in the final study (**Figure 1**). The clinical data and imaging features of the patients in the training cohort and validation cohort are shown in **Table 1**. The 381 patients included 314 men and 67 women with an average age of 55.71 years, ranging from 15 to 80 years. Based on the postoperative histopathological examination, 198 patients presented with MVI. The results of the two radiologists’ evaluation of preoperative imaging features are shown in **Supplementary Table 1**. A diagnosis of each case was made by the two pathologists following the consolidated standards, with good consistency of results (**Supplementary Table 2**). For the evaluation of peritumoral enhancement, the Kappa coefficient of consistency test was more than 0.9, and the Kappa coefficient of evaluation of peritumoral boundary, tumor shape, multiple tumors, MVI status, MVI grade, and tumor differentiation degree was higher than 0.75. The Kappa coefficient of the consistency test among the imaging features was all more beyond 0.70, indicating a good agreement between the two evaluators.

**Table 1:** Clinical and imaging characteristics of HCC patients in the training and validation cohorts.

	training cohort (*n* = 267) no. (%)	validation cohort (*n* = 114) no. (%)	χ^2^	*P*-value
**Age (**years)			0.374	0.541
≥65	59 (22.1)	22 (19.3)		
<65	208 (77.9)	92 (80.7)		
**Gender**			0.078	0.779
Male	221 (82.8)	93 (81.6)		
Female	46 (17.2)	21 (18.4)		
**With hepatitis**			0.414	0.520
Present	220 (82.4)	97 (85.1)		
Absent	47 (17.6)	17 (14.9)		
**With Cirrhosis**			0.014	0.907
Present	207 (77.5)	89 (78.1)		
Absent	60 (22.5)	25 (21.9)		
**AFP** (ng/ml)			0.331	0.565
>400	102 (38.2)	40 (35.1)		
≤400	165 (61.8)	74 (64.9)		
**AST** (U/L)			0.264	0.876
<45	178 (66.7)	76 (66.7)		
45–90 >90	64 (24.0) 25 (9.3)	29 (25.4) 9 (7.9)		
**ALT** (U/L)			0.952	0.621
≤40	158 (59.2)	73 (64.0)		
40–80 >80	76 (28.5) 33 (12.3)	30 (26.3) 11 (9.7)		
**Total bilirubin** (umol/L)			0.261	0.609
>17.1	120 (44.9)	48 (42.1)		
≤17.1	147 (55.1)	66 (57.9)		
**Direct bilirubin** (umol/L)			0.009	0.923
>3.4	198 (74.2)	84 (73.7)		
≤3.4	69 (25.8)	30 (26.3)		
**Total protein** (g/L)			0.741	0.389
≥65	144 (53.9)	56 (49.1)		
<65	123 (46.1)	58 (50.9)		
**Serum albumin** (g/L)			0.024	0.878
≥40	110 (41.2)	46 (40.4)		
<40	157 (58.8)	68 (59.6)		
**Neutrophils** (×10^9^/L)			1.027	0.311
>6.30	33 (12.4)	10 (8.8)		
≤6.30	234 (87.6)	104 (91.2)		
**Platelet **(×10^9^/L)			0.190	0.663
≥125	154 (57.7)	63 (55.3)		
<125	113 (42.3)	51 (44.7)		
**Lymphocyte **(×10^9^/L)			0.002	0.969
≥1.10	154 (57.7)	66 (57.9)		
<1.10	113 (42.3)	48 (42.1)		
**VEGF-A** (pg/ml) ≤138.30 >138.30	140 (52.4) 127 (47.6)	54 (47.4) 60 (52.6)	0.820	0.365
**MVI**			1.661	0.197
Present	133 (49.8)	65 (57.0)		
Absent	134 (50.2)	49 (43.0)		
**Tumor size** (cm)			1.758	0.415
≥5 3–5	138 (51.7) 80 (30.0)	60 (52.6) 39 (34.2)		
<3	49 (18.4)	15 (13.2)		
**Peritumoral enhancement**			0.010	0.921
Present	125 (46.8)	54 (47.4)		
Absent	142 (53.2)	60 (52.6)		
**Peritumoral boundary**			0.002	0.967
Clear boundary	106 (39.7)	45 (39.5)		
Unclear boundary	161 (60.3)	69 (60.5)		
**Tumor shape**			0.382	0.537
Regular shape	105 (39.3)	41 (36.0)		
Irregular shape	162 (60.7)	73 (64.0)		
**Intratumoral artery**			0.003	0.956
Present	128 (47.9)	55 (48.2)		
Absent	139 (52.1)	59 (51.8)		
**Multiple tumors**			0.082	0.775
Present	21 (7.9)	8 (7.0)		
Absent	246 (92.1)	106 (93.0)		

### Serum VEGF-A Was a Predictive Factor of MVI in HCC Patients

**Figure 3:**
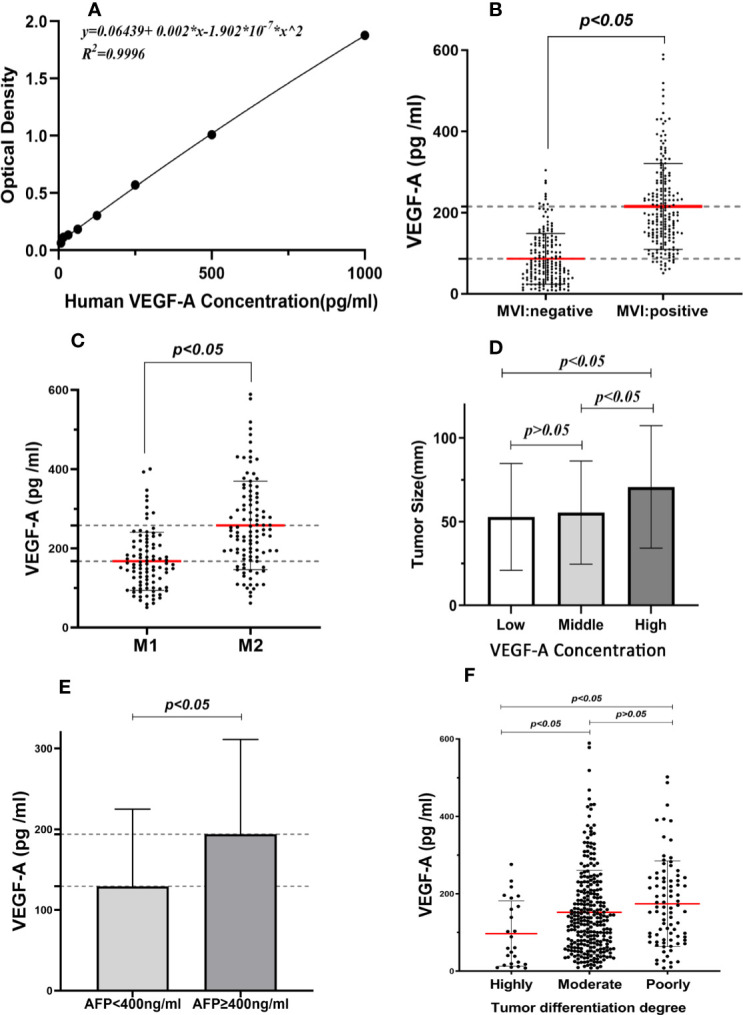
Association between serum VEGF-A expression and clinicopathological factors. **(A)** Standard curve of OD values and the VEGF-A concentration. **(B)** Serum VEGF-A levels in MVI positive group was higher than MVI negative group. **(C)** The mean VEGF-A concentration was significantly difference between M1 group and M2 group. **(D)** The expression of VEGF-A was associated with larger tumor size. **(E)** Pretreatment VEGF-A levels were higher in patients whose preoperative serum AFP >400 ng/ml. **(F)** The highly differentiated tumors expressed lower VEGF-A levels.

A standard curve was constructed for the determination of VEGF-A concentration according to the manufacturer’s instructions (**Figure 3A**). Next, according to the direct relationship between optical density (OD) and the VEGF-A concentration standard curve, we determined the serum VEGF-A concentration of the patients included in the study. The serum VEGF-A levels in the MVI positive group (n = 198) and the MVI negative group (n = 183) were 215.25 ± 105.68 pg/ml and 86.52 ± 62.45pg/ml, respectively (*P <*0.05, **Figure 3B**). In addition, the mean VEGF-A concentration in the M2 group (n = 104) and the M1 group (n = 94) was 258.33 pg/ml and 167.60 pg/ml, respectively (*P <*0.05, **Figure 3C**). According to the data distribution features of VEGF-A concentration, we divided it into three groups, which were low level group (<100 pg/ml), middle level group (100–200 pg/ml), and high level group (>200 pg/ml). The mean tumor size in the three VEGF-A concentration groups was 52.86, 55.43, and 70.77 mm, respectively (**Figure 3D**). Pretreatment VEGF-A levels were much higher in patients whose preoperative serum AFP >400 ng/ml (**Figure 3E**). HCC patients had different degrees of tumor differentiation, and the highly differentiated group exhibited lower VEGF-A expression level (**Figure 3F**).

**Figure 4:**
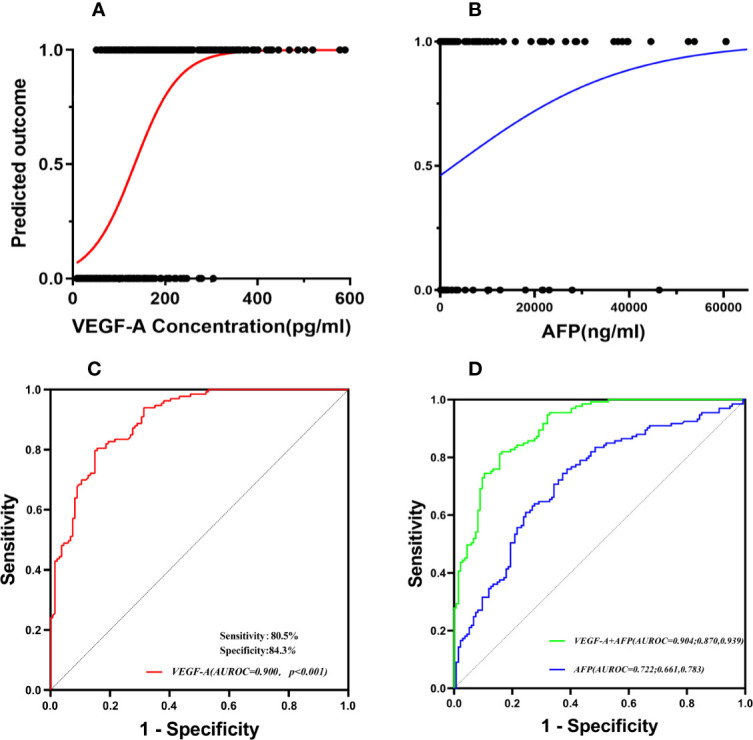
Serum VEGF-A was a predictive factor of MVI in HCC patients. The probability of MVI increased along with the increase of VEGF-A and AFP concentrations **(A, B)**. **(C)** The cutoff value of VEGF-A concentration was determined by receiver operating characteristic curve (ROC) analysis. **(D)** The combined two indicators (VEGF-A and AFP) were more effective than AFP alone in predicting MVI.

The logistic regression analysis found that the probability of MVI increased along with the increase of VEGF-A and AFP concentrations (**Figures 4A, B**). The cutoff value of VEGF-A concentration was determined to be 138.30 pg/ml, according to receiver operating characteristic curve analysis in the training cohort (**Figure 4C**), defined as the threshold value optimally separating the MVI negative patients from the MVI positive patients. This value yielded sensitivity of 80.5 and 70.8% for training cohort and validation cohort, respectively. This value also yielded specificity of 84.3 and 71.4% for the training cohort and validation cohort, respectively. VEGF-A gave a good predictive performance for HCC patients with MVI (AUC: 0.900; 95%CI:0.865–0.935). By using VEGF-A combined with AFP to draw a new ROC curve, the results show that VEGF-A combined with AFP was more effective than AFP alone in predicting MVI in the training cohort (AUROC: 0.904 and 0.722, respectively) (**Figure 4D**).

### Univariate and Multivariate Analyses of Presence of MVI

Univariate logistic regression analysis revealed that AFP, AST, lymphocyte count, and VEGF-A significantly correlated with MVI in **Table 2**. In addition, tumor size, peritumoral enhancement, peritumoral boundary, tumor shape, intratumoral artery, and multiple tumors also associated to MVI. Variables with *P <*0.05 in univariate analysis were included in the multivariate analysis. Besides VEGF-A, the data of blood biomarkers were divided based on a cut-off value of the normal range. The cut-off values of AFP and lymphocyte count were 400 ng/ml and 1.10 × 10^9^/L respectively. Higher serum concentration of AFP and VEGF-A, lower lymphocyte count, peritumoral enhancement, irregular tumor shape, and intratumoral artery were identified as significant predictors for MVI. Compared with the group that VEGF-A ≤138.30 pg/ml, VEGF-A >138.30 pg/ml indicated higher risk of MVI in HCC patients (OR: 33.088; 95%CI: 12.871–85.057; *P <*0.001). Intratumoral artery also had the strong predictive power for the presence of MVI (OR: 7.121; 95%CI: 2.830–17.922).

**Table 2:** Univariate and multivariate logistic regression analysis of prediction of MVI in training cohort.

Characteristics		Univariate Logistic regression analysis		Multivariate Logistic regression analysis	
		OR (95%CI)	*P*-value	OR(95%CI)	*P*-value
**Gender**	Male *vs* Female	1.385 (0.730, 2.626)	0.318	–	
**Age (**years)	≥65 *vs <*65	1.757 (0.974, 3.169)	0.061	–	
**With hepatitis**	Present vs Absent	1.284 (0.682, 2.418)	0.439	–	
**With Cirrhosis**	Present *vs* Absent	1.399 (0.784, 2.496)	0.255	–	
**AFP** (ng/ml)	>400 *vs ≤*400	4.405 (2.581, 7.518)	<0.001	4.327 (1.803, 10.384)	0.001
**AST** (U/L)	<45 45–90 *vs <*45 >90 *vs <*45	Control 2.564 (1.419, 4.633) 4.555 (1.735, 11.958)	<0.001 0.002 0.002	–	0.195
**ALT** (U/L)	<40 40–80 *vs <*40 >80 *vs <*40	Control 1.024 (0.592, 1.770) 1.660 (0.773, 3.567)	0.420 0.933 0.194	–	
**Total bilirubin (**umol/L)	>17.1 *vs ≤*17.1	1.216 (0.750, 1.970)	0.428	—	
**Direct bilirubin (**umol/L)	>3.4 *vs ≤*3.4	1.302 (0.751, 2.258)	0.347	—	
**Total protein** (g/L)	<65 *vs* ≥65	1.461 (0.901, 2.370)	0.124	—	
**Serum albumin** (g/L)	<40 *vs* ≥40	1.077 (0.662, 1.754)	0.764	—	
**Neutrophils (**10^9^/L)	>6.30 *vs ≤*6.30	1.081 (0.521, 2.241)	0.835	—	
**Platelet (**10^9^/L)	<125 *vs* ≥125	1.151 (0.708, 1.871)	0.571	—	
**Lymphocyte (**10^9^/L)	<1.10 *vs* ≥1.10	3.267 (1.965, 5.432)	<0.001	2.747 (1.193, 6.328)	0.018
**VEGF-A** (pg/ml)	>138.30 *vs ≤*138.30	21.125 (11.264, 39.619)	<0.001	33.088 (12.871, 85.057)	<0.001
**Tumor size** (cm)	<3 3–5 *vs <*3 >5 *vs <*3	Control 1.071 (0.490, 2.344) 5.523 (2.687, 11.312)	<0.001 0.863 <0.001	–	0.954
**Peritumoral enhancement**	Present *vs* Absent	7.725 (4.473, 13.341)	<0.001	2.415(1.009, 5.781)	0.048
**Peritumoral boundary**	Clear boundary*vs* Unclear boundary	3.062 (1.832, 5.119)	<0.001	—	0.502
**Tumor shape**	Regular shape *vs* Irregular shape	5.468 (3.173, 9.425)	<0.001	3.504(1.478, 8.307)	0.004
**Intratumoral artery**	Present *vs* Absent	12.508 (7.004, 22.337)	<0.001	7.121(2.830, 17.922)	< 0.001
**Multiple tumors**	Present *vs* Absent	2.712 (1.019, 7.220)	0.046	—	0.084

### Development and Validation of Nomogram for Prediction of MVI

**Figure 5:**
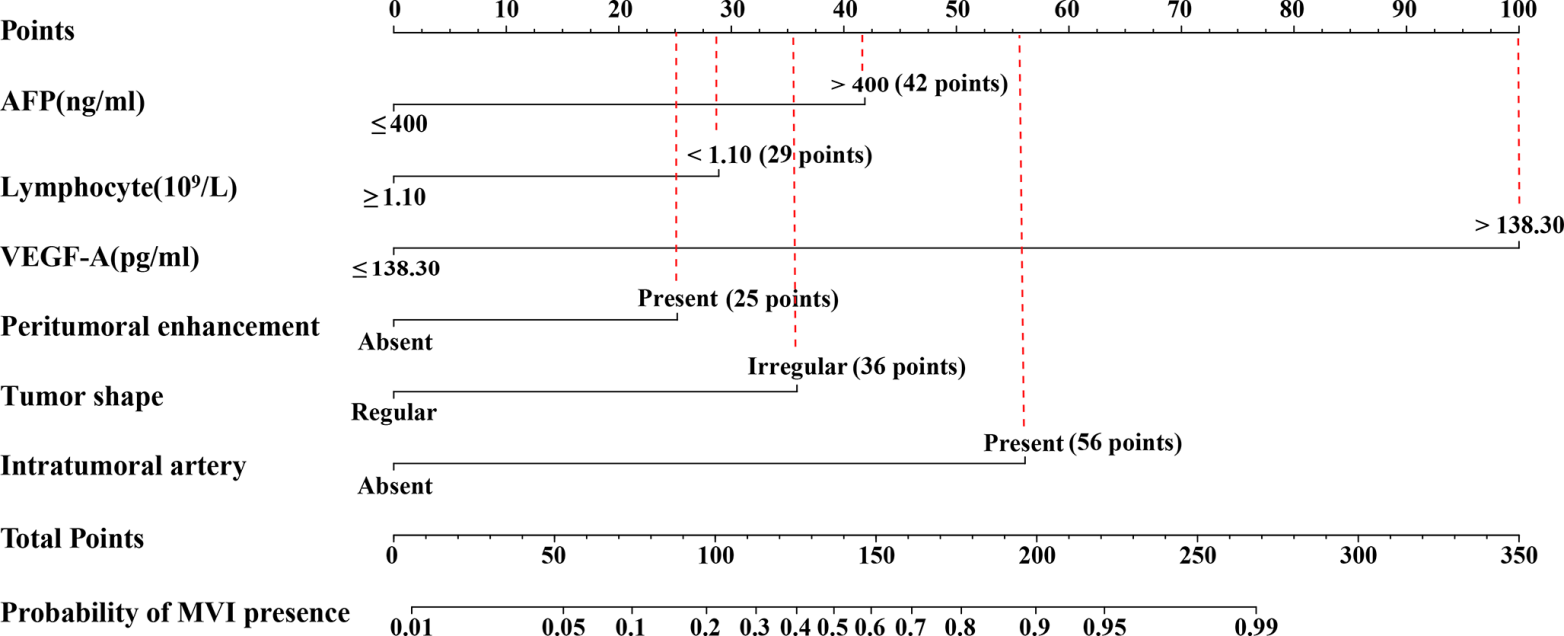
Nomogram to predicting MVI. The use of nomogram was as follows: Points are assigned for each variable by drawing a straight line upward from the corresponding value to the “Points” line. Then, sum the points received for each variable, and locate the number on the “Total Points” axis. To speculate the patient’s MVI status, a straight line must be drawn down to the corresponding “MVI presence” probability axis. AFP, alpha fetoprotein; VEGF-A, Vascular endothelial growth factor A; MVI, microvascular invasion.

**Figure 6:**
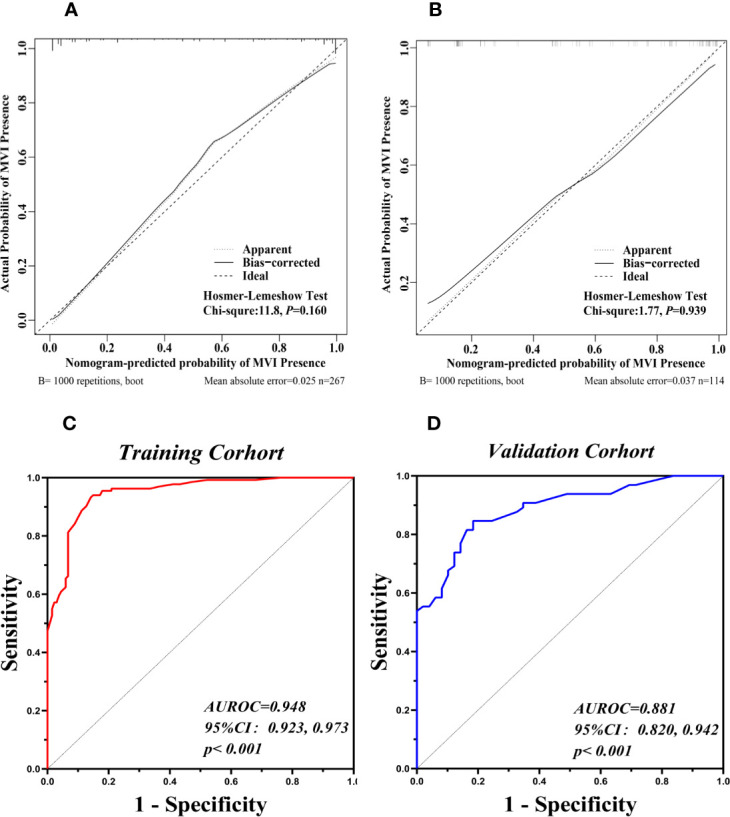
Validation of the Nomograms for prediction of MVI. The calibration curves for predicting presence of MVI in the training cohort **(A)** and the validation cohort **(B)**. Nomogram-predicted MVI presence was plotted on the X-axis, and the actual MVI presence was plotted on the Y-axis. A plot along the 45° line would indicate a perfect calibration model in which the predicted presence are identical to the actual presence. The apparent line represented the fitting line between the predicted situation of the model and the actual situation. The ROC curves of the nomogram in the training cohort (**C,** AUROC: 0.948) and the validation cohort (**D,** AUROC: 0.881).

The nomogram was based on proportionally converting each regression coefficient in multivariate analysis to a 0-to 100- point scale. The effect of the variable with the highest *β* coefficient (absolute value) was assigned 100 points (**Supplementary Table 3**). The points were added across independent variables to derive total points, converted to predicted probabilities of MVI status. The nomogram integrated AFP, VEGF-A, lymphocyte count, and imaging biomarkers accessed by logistic regression (**Figure 5**). Serum VEGF-A indicated the largest effect on the prediction of MVI, with a maximal score of 100 points if VEGF-A >138.30 pg/ml. The calibration curve showed that the prediction results of the nomogram model were in good agreement with histopathologic confirmation in both cohorts. The results of the Hosmer–Lemeshow test showed that there was no statistical difference between prediction outcome of nomogram and actual results in the two cohorts (χ²: 11.80, *P* = 0.160, χ²: 1.77, *P* = 0.939 respectively; **Figures 6A, B**), indicating that the nomogram was effective in the prediction of MVI. To evaluate and compare the discriminatory power of nomogram model, we plotted the receiver operating characteristic (ROC) curve and calculated the area under the ROC curve (AUC) with 95% confidence intervals (CI) in the training cohort and validation cohort, respectively. The area under the ROC curve of the established nomogram in the training cohort and validation cohort was 0.948 (95%CI: 0.923, 0.973) and 0.881(95%CI: 0.820, 0.942) respectively (**Figures 6C, D**).

**Figure 7:**
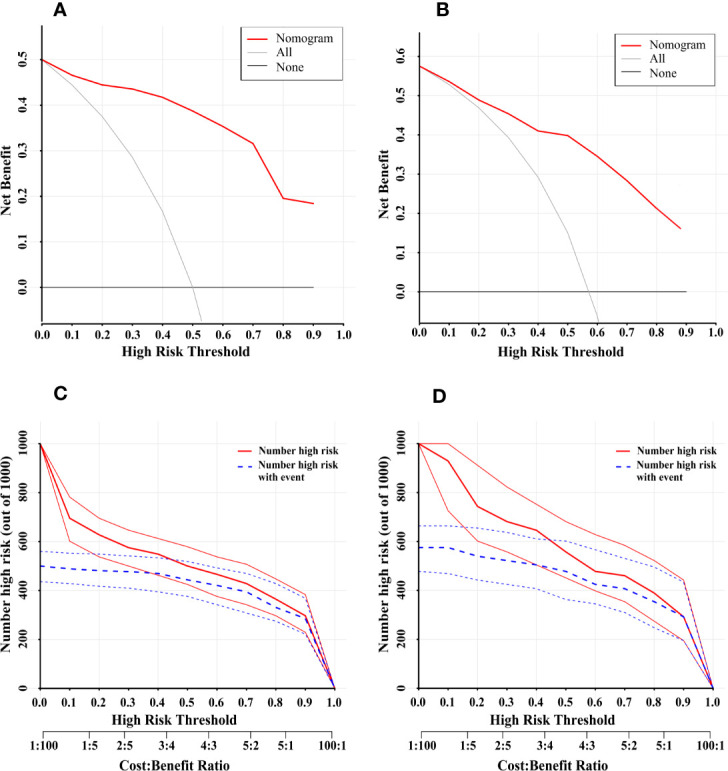
Decision Curve Analysis (DCA) for the nomogram in the training cohort and validation cohort **(A, B)**. The *Y*-axis showed the net benefit. The *X*-axis represented the threshold probability. The None-line (black line) represented the net benefit when none of the participants were considered to have MVI. The All-line (light gray line) represented the net benefit when all participants were considered to have MVI. The red line represented the net benefit of the nomogram at different threshold probabilities. The area between the “None-line” (black line) and “All-line” (light gray line) in the model curve indicated the clinical utility of the model. Clinical Impact Curve (CIA) of the nomogram for predicting presence of MVI in the training cohort **(C)** and validation cohort **(D)**. At different threshold probabilities within a given population, the number of high-risk patients and the number of high-risk patients with MVI were shown.

The cutoff value was set at 127 according to the ROC curve in the training cohort. The sensitivity and specificity were 94.0 and 85.1% in the training cohort and 78.5 and 75.5% in the validation cohort, respectively. The positive predictive value, negative predictive value, and diagnostic accuracy of the nomogram model were 86.2, 94.4, and 89.5% in the training cohort, respectively. The positive predictive value, negative predictive value, and diagnostic accuracy were 81.0, 72.5, and 77.2% in the validation cohort, respectively. The DCA revealed that using the nomogram to predict MVI would probably add more benefit than treating either all or no patients in training and validation cohort (**Figures 7A, B**).Clinical impact curve found that the predictive power of the nomogram model was remarkable (**Figures 7C, D**).

## Discussion

MVI is a well-known risk factor for early recurrence and poor prognosis of HCC (27). In our study, MVI was present in 52% HCC patients who underwent radical resection. MVI is usually histopathologically diagnosed, and the gold standard to determine MVI statues is postoperative pathology examination of surgical specimens. Hollow core needle biopsy is not recommended to diagnose MVI before operation as this method has not met ideal sensitivity (28). In addition, there is a risk of needle tract dissemination during the procedure of hollow core needle biopsy (29). Given the important role of MVI on treatment options, it is necessary to predict the presence of MVI preoperatively. In the present study, we found AFP >400 ng/ml, lymphocyte count <1.10 × 10^9^/L, VEGF-A ≥138.30 pg/ml, peritumoral enhancement, irregular tumor shape, and intratumoral artery were significantly associated with MVI. Interestingly, there was no previous study that indicated the low lymphocyte count is a preoperative risk factor for MVI (OR: 2.747; 95%CI: 1.193–6.328). Lymphocyte depletion reflects an impaired T lymphocyte-mediated antitumor response (30), and tumor cells may give priority to deplete regulatory T cells in the process of progression (31, 32). Lymphocyte depletion in advanced HCC may provide an explanation for a low lymphocyte level in MVI positive patients.

Comparing MRI and ultrasound imaging, contrast-enhanced CT examination is recognized as an effective, readily accessible, and inexpensive tool for assessing MVI status. Three imaging features (peritumoral enhancement, irregular tumor shape, and intratumoral artery) were found as the independent predictors for MVI presence in the present study. Lin et al. pointed that the CT signs of hepatocellular carcinoma with MVI showed peritumoral area enhanced in the arterial phase, and enhancement disappeared in the subsequent stage (33). The stark contrast area may be the area of decrease or absent portal venous flow (14). At the same time, the formation of intratumoral artery and peritumor enhancement may be the signs of compensatory arterial hyper-perfusion caused by microvascular tumor thrombus blocking the small branches of hepatic vein (34). Our results were in line with the previous study (35), which found that irregular tumor shape was a high risk factor for MVI in HCC. In the present study, we found that tumor diameter >5 cm and unclear tumor boundary were not related to the occurrence of MVI by multivariate analysis, which was different with the previous study (36).

Angiogenesis is a critical event during microvascular invasion (37), where VEGF-A as a key regulatory factor in tumor angiogenesis (18, 38) was found significantly associated with MVI in the present study. By measuring the VEGF-A concentration, we found the probability of MVI increased along with the increase of VEGF-A. Compared with serum AFP levels, serum VEGF-A provided a higher accuracy for predicting MVI. Our study went one step further in that VEGF-A levels were much higher in patients whose preoperative serum: AFP >400 ng/ml compared to AFP ≤400 ng/ml. Moreover, to our knowledge, it was the first time that VEGF-A was introduced to develop a non-invasive MVI nomogram.

By combining blood biomarkers and enhanced CT features, we established a multivariable nomogram with strong predictive capacity. It represented excellent prediction accuracy than single radiological indicator models. The AUROC in the training cohort was 0.948 (95%CI: 0.923, 0.973), and the AUROC in the validation cohort was 0.881 (95%CI: 0.820, 0.942). The nomogram model exhibited good distinction and calibration, which could be used to diagnose MVI or to identify high-risk patients with MVI before operation. One of the greatest advantages of the nomogram model in the present study was that all the parameters incorporated were easily acquired, so that the model could be easily applied in clinical practice.

However, the present study had limitations. First, the lack of external validation was the major limitation, and expanding the study results to other medical centers may be required to validate its reproducibility. Second, in the radiographic study, we only referred to enhanced CT, without comprehensive use of ultrasound, MRI, and other imaging methods, so there is a potential bias in imaging evaluation. Third, the nomogram may have difficulty to detect atypical signs of MVI lesion. Finally, because of its retrospective character, a large number of patients who are clinically deemed ‘‘high-risk” for MVI but who did not undergo surgical management were excluded.

## Conclusion

Higher serum concentrations of AFP and VEGF-A, lower lymphocyte count, peritumoral enhancement, irregular tumor shape, and intratumoral artery are promising markers for MVI prediction in HCC. The nomogram incorporated VEGF-A, AFP, lymphocyte count, and imaging risk factors achieved desirable effectiveness in preoperatively predicting MVI.

## Data Availability Statement

The raw data supporting the conclusions of this article will be made available by the authors, without undue reservation.

## Ethics Statement

The studies involving human participants were reviewed and approved by the Research Ethics Committee of the First Affiliated Hospital of Xi’an Jiaotong University.The patients/participants provided their written informed consent to participate in this study.

## Author Contributions

QL and SH interpreted the study design. QL supervised our study. QL and SH obtained the research fund. HW and SH screened the publications, performed statistics, and drafted the manuscript. YL helped perform statistics. RL and LW screened the publications. All authors contributed to the article and approved the submitted version.

## Funding

This study was supported by grants from the National Natural Science Foundation of China (Nos. 81602566 and 81874069), and the Fundamental Research Funds for the Central Universities (xzy012020050).

## Conflict of Interest

The authors declare that the research was conducted in the absence of any commercial or financial relationships that could be construed as a potential conflict of interest.

## Publisher’s Note

All claims expressed in this article are solely those of the authors and do not necessarily represent those of their affiliated organizations, or those of the publisher, the editors and the reviewers. Any product that may be evaluated in this article, or claim that may be made by its manufacturer, is not guaranteed or endorsed by the publisher.

## Supplementary Material

Supplementary Figure 1 The schematic diagram of sampling sites in liver tumor specimens. **(A–D)** represent the transition area between tumor and adjacent liver tissues at 12, 3, 6, and 9 o’clock. **(E)** represents the tumor tissue. **(F)** represents adjacent liver tissue within 1.0 cm to the tumor. **(G)** represents adjacent liver tissue 1.0 cm away from tumor. One to two pieces are sampled at **(A–D)** and the ratio of tumor and adjacent liver tissue is 1:1. Two to three pieces are sampled at **(E)** Thirdly, one to two pieces are sampled at **(F)** Lastly, one to two pieces are sampled at **(G)** if the surgical margin is wide enough. Each piece is marked, and the volume is about (1.0–2.0) cm × 1.0 cm × 0.2 cm.
