# Supplementary figures and images for: A Non-Invasive Nomogram for Preoperative Prediction of Microvascular Invasion Risk in Hepatocellular Carcinoma
Source: Front Oncol. 2021 Dec 24;11:745085. doi: 10.3389/fonc.2021.745085 (PMC8739965; doi:10.3389/fonc.2021.745085)

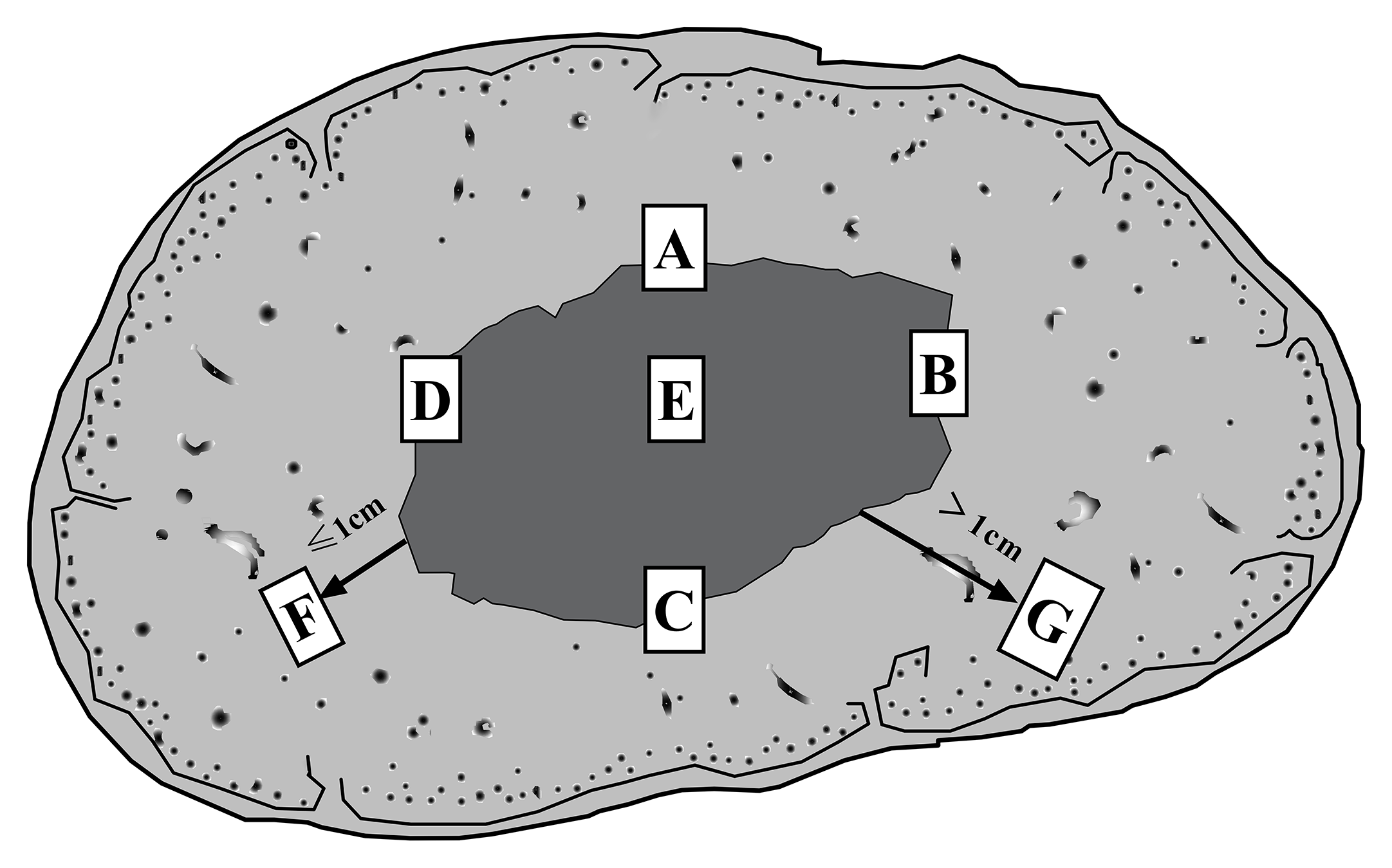

Supplement: Supplementary Figure 1 — The schematic diagram of sampling sites in liver tumor specimens. (A–D) represent the transition area between tumor and adjacent liver tissues at 12, 3, 6, and 9 o’clock. (E) represents the tumor tissue. (F) represents adjacent liver tissue within 1.0 cm to the tumor. (G) represents adjacent liver tissue 1.0 cm away from tumor. One to two pieces are sampled at (A–D) and the ratio of tumor and adjacent liver tissue is 1:1. Two to three pieces are sampled at (E) Thirdly, one to two pieces are sampled at (F) Lastly, one to two pieces are sampled at (G) if the surgical margin is wide enough. Each piece is marked, and the volume is about (1.0–2.0) cm × 1.0 cm × 0.2 cm. [file Image_1.tif]
