# Supplementary material for: A Non-Invasive Nomogram for Preoperative Prediction of Microvascular Invasion Risk in Hepatocellular Carcinoma
Source: Front Oncol. 2021 Dec 24;11:745085. doi: 10.3389/fonc.2021.745085 (PMC8739965; doi:10.3389/fonc.2021.745085)
Supplement: Supplementary file 2 [file Table_1.docx]

**Supplementary information**

**Supplementary TABLE 1∣**The consistency test results of the evaluation of imaging features between the two radiologists.

|  | Radiologist A | Radiologist B | Cohen’s Kappa | 95% CI |
| --- | --- | --- | --- | --- |
| **Peritumoral enhancement**  Present  Absent  **Peritumoral boundary**  Clear boundary  Unclear boundary  **Tumor shape**  Regular shape  Irregular shape  **Intratumoral artery**  Present  Absent  **Multiple tumors**  Present  Absent | 183  198  135  246  146  235  191  190  29  352 | 187  194  138  243  149  232  201  180  44  337 | 0.905  0 .789  0.884  0.716  0.774 | 0.862-0.948  0.724-0.854  0.835 -0.933  0.645-0.787  0.664-0.884 |

**Supplementary TABLE 2∣**The consistency test results of the evaluation of pathological features between the two pathologists.

|  | Pathologist A | Pathologist B | Cohen’s Kappa | 95% CI |
| --- | --- | --- | --- | --- |
| **MVI Status** |  |  | 0.874 | 0.923-0.825 |
| Present | 197 | 203 |  |  |
| Absent | 184 | 178 |  |  |
| **MVI Grade** |  |  | 0.831 | 0.880-0.782 |
| M0 | 184 | 178 |  |  |
| M1 | 96 | 94 |  |  |
| M2 | 101 | 109 |  |  |
| **Tumor differentiation degree** |  |  | 0.877 | 0.928-0.826 |
| Highly-differentiated | 30 | 26 |  |  |
| Moderate-differentiated | 266 | 273 |  |  |
| Poorly-differentiated | 85 | 82 |  |  |

**Supplementary TABLE 3∣** Multivariate logistic regression analysis of MVI presence based on preoperative data in the training cohort

| **Variables** | ***β* coefficient**  **(absolute value)** | **OR(95%CI)** | ***P*-value** | **points assigned** |
| --- | --- | --- | --- | --- |
| **AFP** > **400** | 1.465 | 4.327(1.803,10.384) | 0.001 | 42 |
| **Lymphocyte < 1.10× 10^9/L** | 1.011 | 2.747(1.193，6.328） | 0.018 | 29 |
| **VEGF-A** > **138.30 pg/ml** | 3.499 | 33.088(12.871, 85.057) | < 0.001 | 100 |
| **Peritumoral enhancement** | 0.882 | 2.415(1.009, 5.781) | 0.048 | 25 |
| **Tumor shape** | 1.254 | 3.504(1.478, 8.307) | 0.004 | 36 |
| **Intratumoral artery** | 1.963 | 7.121(2.830, 17.922) | < 0.001 | 56 |
